# Machine learning algorithms for identifying predictive variables of mortality risk following dementia diagnosis: a longitudinal cohort study

**DOI:** 10.1038/s41598-023-36362-3

**Published:** 2023-06-10

**Authors:** Shayan Mostafaei, Minh Tuan Hoang, Pol Grau Jurado, Hong Xu, Lluis Zacarias-Pons, Maria Eriksdotter, Saikat Chatterjee, Sara Garcia-Ptacek

**Affiliations:** 1 Division of Clinical Geriatrics, Department of Neurobiology, Care Sciences and Society, Karolinska Institute, Stockholm, Sweden; 2 Department of Medical Epidemiology and Biostatistics, Karolinska Institute, Stockholm, Sweden; 3 Vascular Health Research Group of Girona (ISV-Girona), Institut Universitari d’Investigació en Atenció Primària Jordi Gol i Gurina (IDIAP Jordi Gol), Girona, Spain; 4 Network for Research on Chronicity, Primary Care, and Health Promotion (RICAPPS), Tenerife, Spain; 5 Aging and Inflammation Theme, Karolinska University Hospital, Stockholm, Sweden; 6 Division of Information Science and Engineering, School of Electrical Engineering and Computer Science, KTH Royal Institute of Technology, Stockholm, Sweden

**Keywords:** Statistics, Machine learning, Neurological disorders

## Abstract

Machine learning (ML) could have advantages over traditional statistical models in identifying risk factors. Using ML algorithms, our objective was to identify the most important variables associated with mortality after dementia diagnosis in the Swedish Registry for Cognitive/Dementia Disorders (SveDem). From SveDem, a longitudinal cohort of 28,023 dementia-diagnosed patients was selected for this study. Sixty variables were considered as potential predictors of mortality risk, such as age at dementia diagnosis, dementia type, sex, body mass index (BMI), mini-mental state examination (MMSE) score, time from referral to initiation of work-up, time from initiation of work-up to diagnosis, dementia medications, comorbidities, and some specific medications for chronic comorbidities (e.g., cardiovascular disease). We applied sparsity-inducing penalties for three ML algorithms and identified twenty important variables for the binary classification task in mortality risk prediction and fifteen variables to predict time to death. Area-under-ROC curve (AUC) measure was used to evaluate the classification algorithms. Then, an unsupervised clustering algorithm was applied on the set of twenty-selected variables to find two main clusters which accurately matched surviving and dead patient clusters. A support-vector-machines with an appropriate sparsity penalty provided the classification of mortality risk with accuracy = 0.7077, AUROC = 0.7375, sensitivity = 0.6436, and specificity = 0.740. Across three ML algorithms, the majority of the identified twenty variables were compatible with literature and with our previous studies on SveDem. We also found new variables which were not previously reported in literature as associated with mortality in dementia. Performance of basic dementia diagnostic work-up, time from referral to initiation of work-up, and time from initiation of work-up to diagnosis were found to be elements of the diagnostic process identified by the ML algorithms. The median follow-up time was 1053 (IQR = 516–1771) days in surviving and 1125 (IQR = 605–1770) days in dead patients. For prediction of time to death, the CoxBoost model identified 15 variables and classified them in order of importance. These highly important variables were age at diagnosis, MMSE score, sex, BMI, and Charlson Comorbidity Index with selection scores of 23%, 15%, 14%, 12% and 10%, respectively. This study demonstrates the potential of sparsity-inducing ML algorithms in improving our understanding of mortality risk factors in dementia patients and their application in clinical settings. Moreover, ML methods can be used as a complement to traditional statistical methods.

## Introduction

Dementia is a major and growing public health concern with a substantial increase in prevalence projected in the future^1,2^. Mortality risk is higher in patients with dementia (PWD) compared to dementia-free subjects^3^. Risk factors such as age, Body Mass Index (BMI), sex, Mini-Mental State Examination (MMSE) score, and comorbidities are significantly associated with mortality risk in dementia patients^4–9^. These patients have common comorbidities for which treatment could be a critical determinant of survival^10,11^. Despite decades of research, there may be factors related to mortality risk in dementia which remain undiscovered to date. The identification of these additional risk factors related to death in dementia and understanding of their prognostic role is essential for life and health-care planning and patient care^12^.

Regularized Machine learning (ML) can handle large-scale data and, if properly trained, can give accurate results, especially in a sparse model^13^. ML algorithms could be applied for prediction of mortality risk and time to death and contribute to our understanding of risk factors and their interactions during dementia progression^14^. ML studies can give more accurate results than traditional statistical models since they offer more flexible alternatives in handling large-scale and heterogeneous data^15^. From a clinical viewpoint, achieving a high prediction accuracy in and of itself is not the primary goal. Rather, discovering the most important risk factors is often the primary clinical question. There are several ML strategies to develop time to death models. A recent study showed that Boosted Cox regression outperformed Cox proportional hazards and random forest algorithms for early detection and tracking of Alzheimer's disease^16^.

Our previous studies using traditional statistical methods (e.g., Cox- proportional-hazards model) on the Swedish Registry for Cognitive/Dementia Disorders (SveDem) showed that age, sex, residency, population density, comorbidity burden, BMI, MMSE score, number of medications used and certain specific medications were significantly associated with time to death^6–8,10,11,17,18^. A limitation to such traditional statistical methods is that they do not identify the most important variables among all relevant variables, relying rather on a pre-existing suspicion of linear associations or hypothesis and testing only those associations. The ML algorithms can select a sub-set of the variables, rank them in order of importance (e.g., Gini coefficient) and then identify associations that were not suspected according to a priori* hypothesis*. The aim of this study was to identify variables associated with mortality risk in PWD and rank them in order of importance using sparsity-inducing ML classifiers. The aim was also to develop multivariable models using these variables of mortality risk and time to death. We also compared the predictive power of the classifiers to find the best model for predicting mortality risk in dementia.

## Results

### Patients characteristics

Among 28,023 patients, 16,273 (58.07%) were women, and the mean age and BMI at the time of dementia diagnosis were 78.6 (SD = 7.85) years and 24.8 (SD = 4.39), respectively. The most common dementia type was Alzheimer's disease (AD) 14,464 (51.61%, including early and late AD). The median MMSE score was 22.0 (Interquartile range (IQR) = 18–25) at the time of the diagnosis. The median time from referral to initiation of work-up and from initiation of work-up to diagnosis were 29 (IQR = 14–56) days and 57 (IQR = 26–100) days, respectively. Cardiovascular disease, cancer, and depression were the most frequent comorbidities, present in 69%, 34.1%, and 28.42%, respectively. The median number of medications taken by the patients at the time of the diagnosis was 2 (IQR = 1–2). Additionally, cholinesterase inhibitors, statins, diuretics, and antidepressants were prescribed in 47.51%, 34.11%, 28.71%, and 27.1% of the included patients, respectively. A total of 66.29% (n = 18,576) patients had died by December 31, 2018. The median follow-up time was 1053 (IQR = 516–1771) days in surviving and 1125 (IQR = 605–1770) days in dead patients (Table 1).

**Table 1:** Patient’s characteristics.

Characteristics	Unit/level	Total (N = 28,023)	Survivor (N = 9447)	Dead (N = 18,576)
Age at diagnosis	Years	78.63 ± 7.85	75.53 ± 8.05	80.21 ± 7.26
Sex	Male	11,750 (41.92%)	3,561 (37.7%)	8189 (44.1%)
BMI	Points	24.85 ± 4.38	25.40 ± 4.43	24.58 ± 4.34
Dementia type	Early Alzheimer’s disease (AD)	7140 (25.47%)	2108 (22.31%)	5032 (27.08%)
	Late Alzheimer’s disease (AD)	7324 (26.13%)	2,079 (22%)	5245 (28.23%)
	Mixed dementia in AD and vascular	6,725 (24%)	2,581 (27.32%)	4414 (22.30%)
	Vascular dementia	3044 (10.86%)	549 (5.81%)	2495 (13.43%)
	Other dementias	3790 (13.52%)	2,130 (22.54%)	1660 (8.93%)
Type of diagnostic unit	Primary care	22,920 (81.79%)	8,103 (85.77%)	14,817 (79.76%)
	Memory clinic	5103 (18.21%)	1,344 (14.23%)	3759 (20.24%)
MMSE Score	Points	22 (18–25)	23 (20–26)	21 (18–24)
Time from referral to initiation of work-up	Days	29 (14–56)	29 (16–56)	29 (13–56)
Time from initiation of work-up to diagnosis	Days	57 (26–100)	65 (35–111)	55 (21–94)
Physiotherapist assessment	Yes	1719 (6.13%)	370 (3.91%)	1349 (7.26%)
Basic dementia diagnostic work-up	Number of tests	2 (0–2)	2 (1–2)	2 (0–2)
Charlson comorbidity index	Score	2 (1–3)	1 (1–3)	2 (1–3)
Diabetes mellitus	Yes	4143 (14.8%)	1195 (12.6%)	2948 (15.9%)
Cancer	Yes	9551 (34.1%)	3015 (31.9%)	6536 (35.2%)
Cardiovascular disease	Yes	19,341 (69.01%)	6278 (66.45%)	13,063 (70.32%)
Depression	Yes	7964 (28.42%)	2645 (28%)	5329 (28.69%)
Atrial fibrillation	Yes	5044 (18%)	1328 (14.05%)	3882 (20.9%)
Renal disease	Yes	683 (2.44%)	125 (1.32%)	558 (3%)
Acute Kidney Injury	Yes	329 (1.17%)	78 (0.82%)	251 (1.3%)
Anemia	Yes	1175 (4.19%)	300 (3.17%)	875 (4.71%)
Heart failure	Yes	3407 (12.16%)	1083 (11.46%)	2324 (12.51%)
Alcohol related diagnosis	Yes	991 (3.53%)	412 (4.36%)	579 (3.12%)
Liver failure	Yes	250 (0.89%)	79 (0.83%)	171 (0.92%)
Total number of medications at the time of diagnosis	Number	2 (1–3)	2 (1–2)	2 (1–3)
Calcium channel blockers	Yes	6542 (23.34%)	2120 (22.44%)	4422 (23.8%)
Diuretics	Yes	8046 (28.71%)	1861 (19.7%)	6185 (33.3%)
Statins	Yes	9561 (34.11%)	3388 (35.86%)	6173 (33.23%)
Antidepressants	Yes	7595 (27.1%)	2620 (27.73%)	4975 (26.78)
Cholinesterase inhibitors	Yes	13,315 (47.51%)	5153 (54.54%)	8162 (43.93%)
Memantine	Yes	3,188 (11.37%)	1117 (11.83%)	2053 (11.05%)
Antipsychotics	Yes	1710 (6.1%)	565 (5.98%)	1142 (6.15%)

Continuous variables are presented as mean ± SD or median (IQR: Q1-Q3), Categorical variables are presented as N (%).

### Variable selection and model development

**Figure 1:**
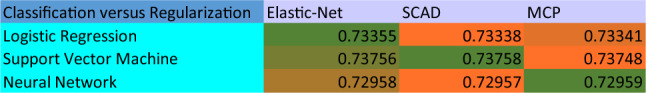
Heatmap of AUROC values for combinations of feature selection methods and classifiers. The heatmap rows represent three classifiers, whereas the columns depict strategies variable selection/regularization methods.

**Figure 2:**
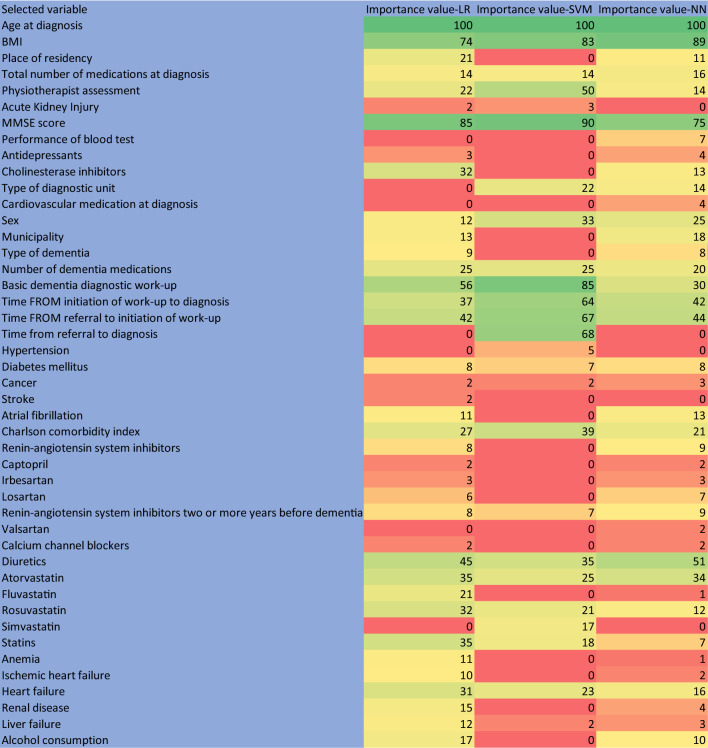
Heatmap for 45 selected variables based on their normalized importance value (%) in each classifier, combined with the three sparsity-inducing penalties. From these variables, twenty variables were consistently identified by all three algorithms.

After adjusting the effect of the follow up time by the statistical control strategy (i.e., including the confounder as control variable in the model), each classifier was combined with the three sparsity-inducing penalties (i.e., Elastic-net, SCAD, and MCP penalties). Then, we tested the combinations of the three sparsity-inducing penalties and three standard classifiers which resulted in nine different combinations to find the best performing models. The heatmap of the area under receiver operating characteristic curve (AUROC) values for each combination of the feature selection methods and the classifiers is shown in Fig. 1. As illustrated, logistic regression (LR) with Elastic-net sparsity penalty, support vector machines (SVM) with the smoothly clipped absolute deviation (SCAD) sparsity penalty, and the combination of backpropagation neural network (NN) with the minimax concave penalty (MCP) resulted in the highest performance with AUROC of 0.7336, 0.7376, and 0.7296, respectively. Based on the results of the best algorithmic combinations of binary classifiers and sparsity-inducing penalties, 38 associated variables were consistently selected and ranked by their importance value using the Elastic net-LR. Twenty-five associated variables were identified by the SCAD-SVM, and 40 variables were consistently identified by the MCP-NN algorithm (Table 2). Finally, 20 variables were consistently selected by all three algorithms (Fig. 2). Among these 20 variables, age at diagnosis, MMSE score, BMI, performance of basic dementia diagnostic work-up, time from referral to initiation of work-up, time from initiation of work-up to diagnosis, and diuretics in the year preceding diagnosis had the highest importance value in prediction of mortality risk across the classifiers (100%, 90%, 89%, 85%, 67%, 64%, and 51%, respectively).

**Table 2:** Selected variables to predict mortality risk based on the training set (N = 18,682) using Elastic-net logistic regression (“glmnet” R package), SCAD- support vector machine (“penalizedSVM” R package), and MCP- neural network algorithm with repeated tenfold cross-validation in the training set (“neuralnet” and “ncvreg” R packages).

Algorithm	Selected variables	Normalized importance value %	*P*-value
Elastic net-regularized logistic regression (Alpha hyperparameter:0.5, lambda tuning’s parameter:0.006196)	Age at dementia diagnosis	100	< 0.001
	MMSE score	85	< 0.001
	BMI	74	< 0.001
	Basic dementia diagnostic work-up	56	< 0.001
	Diuretics	45	< 0.001
	Time from referral to initiation of work-up	42	0.367
	Time from initiation of work-up to diagnosis	37	< 0.001
	Atorvastatin	35	< 0.001
	Statins	35	< 0.001
	Cholinesterase inhibitors	32	< 0.001
	Rosuvastatin	32	< 0.001
	Heart failure	31	0.012
	Charlson comorbidity index	27	< 0.001
	Dementia medications	25	< 0.001
	Physiotherapist assessment	22	< 0.001
	Place of residency	21	0.001
	Fluvastatin	21	0.421
	Alcohol related diagnosis	17	< 0.001
	Renal disease	15	< 0.001
	Total number of medications at baseline	14	< 0.001
	Municipality	13	0.626
	Sex	12	< 0.001
	Liver failure	12	0.062
	Atrial fibrillation	11	< 0.001
	Anemia	11	< 0.001
	Ischemic heart failure	10	< 0.001
	Dementia type	9	< 0.001
	Diabetes mellitus	8	< 0.001
	Renin-angiotensin system inhibitors	8	0.635
	Renin-angiotensin system inhibitors two or more years before dementia diagnosis	8	0.449
	Losartan	6	0.001
	Antidepressants	3	0.135
	Irbesartan	3	0.78
	Acute Kidney Injury	2	0.003
	Cancer	2	0.002
	Stroke	2	< 0.001
	Captopril	2	0.038
	Calcium channel blockers	2	0.367
SCAD-regularized SVM (Kernel: sigmoid, Number of Support Vectors: 18,673, lambda tuning’s parameter:0.010)	Age at dementia diagnosis	100	< 0.001
	MMSE score	90	< 0.001
	Basic dementia diagnostic work-up	85	< 0.001
	BMI	83	< 0.001
	Time from referral to diagnosis	68	< 0.001
	Time from referral to initiation of work-up	67	0.367
	Time from initiation of work-up to diagnosis	64	< 0.001
	Physiotherapist assessment	50	< 0.001
	Charlson comorbidity index	39	< 0.001
	Diuretics	35	< 0.001
	Sex	33	< 0.001
	Dementia medications	25	< 0.001
	Atorvastatin	25	< 0.001
	Heart failure	23	0.012
	Care unit (primary care vs specialist care)	22	< 0.001
	Rosuvastatin	21	< 0.001
	Statins	18	< 0.001
	Simvastatin	17	0.722
	Total number of medications at baseline	14	< 0.001
	Diabetes mellitus	7	< 0.001
	Renin-angiotensin system inhibitors two or more years before dementia diagnosis	7	0.449
	Hypertension	5	< 0.001
	Acute Kidney Injury	3	0.003
	Cancer	2	0.002
	Liver failure	2	0.062
MCP-regularized Backpropagation Neural Network (Softmax activation function, lambda tuning’s parameter:0.0020)	Age at dementia diagnosis	100	< 0.001
	BMI	89	< 0.001
	MMSE score	75	< 0.001
	Diuretics	51	< 0.001
	Time from referral to initiation of work-up	44	0.367
	Time from initiation of work-up to diagnosis	42	< 0.001
	Atorvastatin	34	< 0.001
	Basic dementia diagnostic work-up	30	< 0.001
	Sex	25	< 0.001
	Charlson comorbidity index	21	< 0.001
	Dementia medications	20	< 0.001
	Municipality	18	0.626
	Total number of medications at baseline	16	< 0.001
	Heart failure	16	0.012
	Physiotherapist assessment	14	< 0.001
	Care unit (primary care vs specialist care)	14	< 0.001
	Cholinesterase inhibitors	13	< 0.001
	Atrial fibrillation	13	< 0.001
	Rosuvastatin	12	< 0.001
	Place of residency	11	0.001
	Alcohol related diagnosis	10	< 0.001
	Renin-angiotensin system inhibitors	9	0.635
	Renin-angiotensin system inhibitors two or more years before dementia diagnosis	9	0.449
	Dementia type	8	< 0.001
	Diabetes mellitus	8	< 0.001
	Blood tests	7	0.902
	Losartan	7	0.001
	Statins	7	< 0.001
	Antidepressants	4	0.135
	Cardiovascular medication at diagnosis	4	< 0.001
	Renal disease	4	< 0.001
	Cancer	3	0.002
	Irbesartan	3	0.78
	Liver failure	3	0.062
	Captopril	2	0.038
	Valsartan	2	0.096
	Calcium channel blockers	2	0.367
	Heart failure	2	0.359
	Fluvastatin	1	0.421
	Anemia	1	< 0.001

*P*-values were reported using simple logistic regression, importance values were reported based on Gini index.

### Model performance and comparisons of predictive power

**Figure 3:**
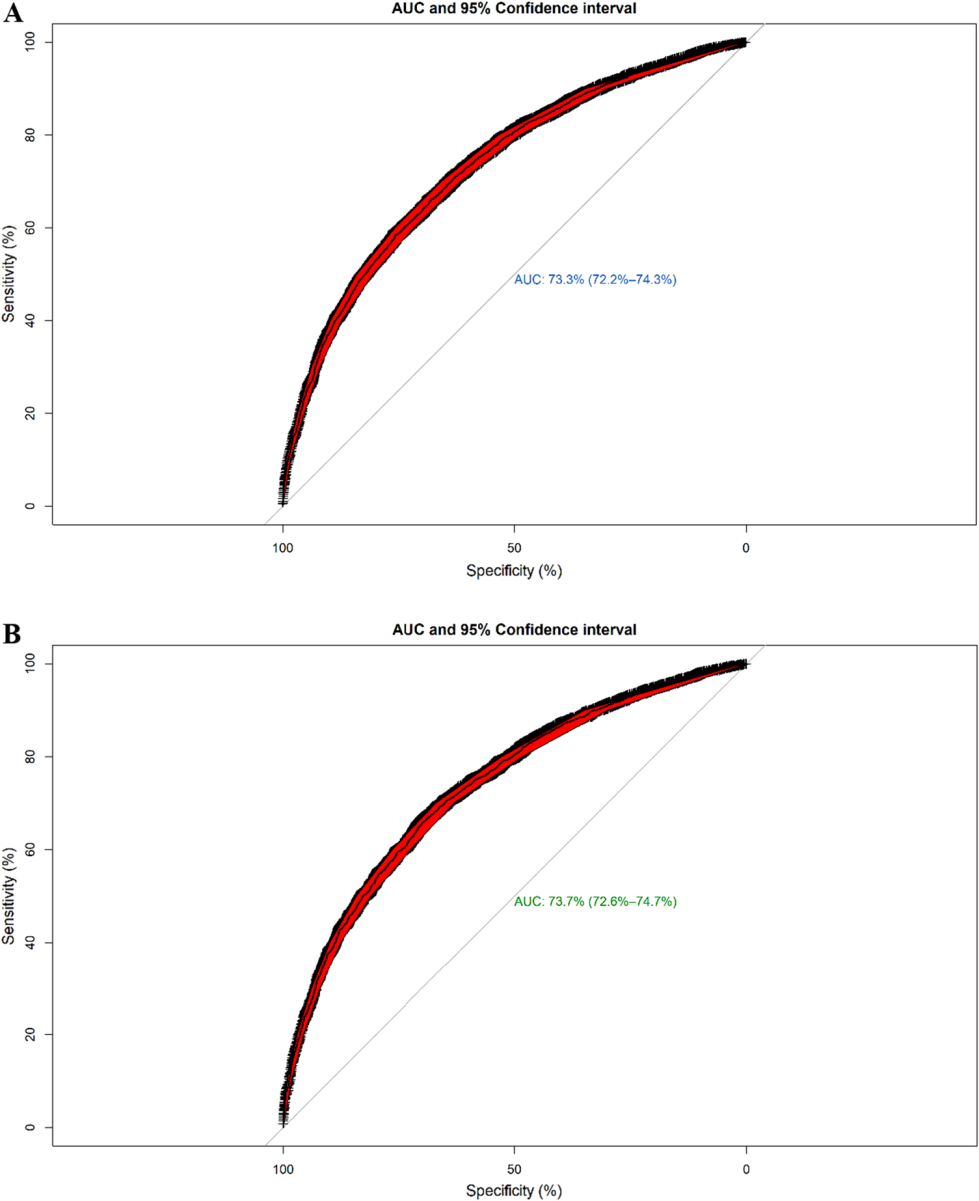

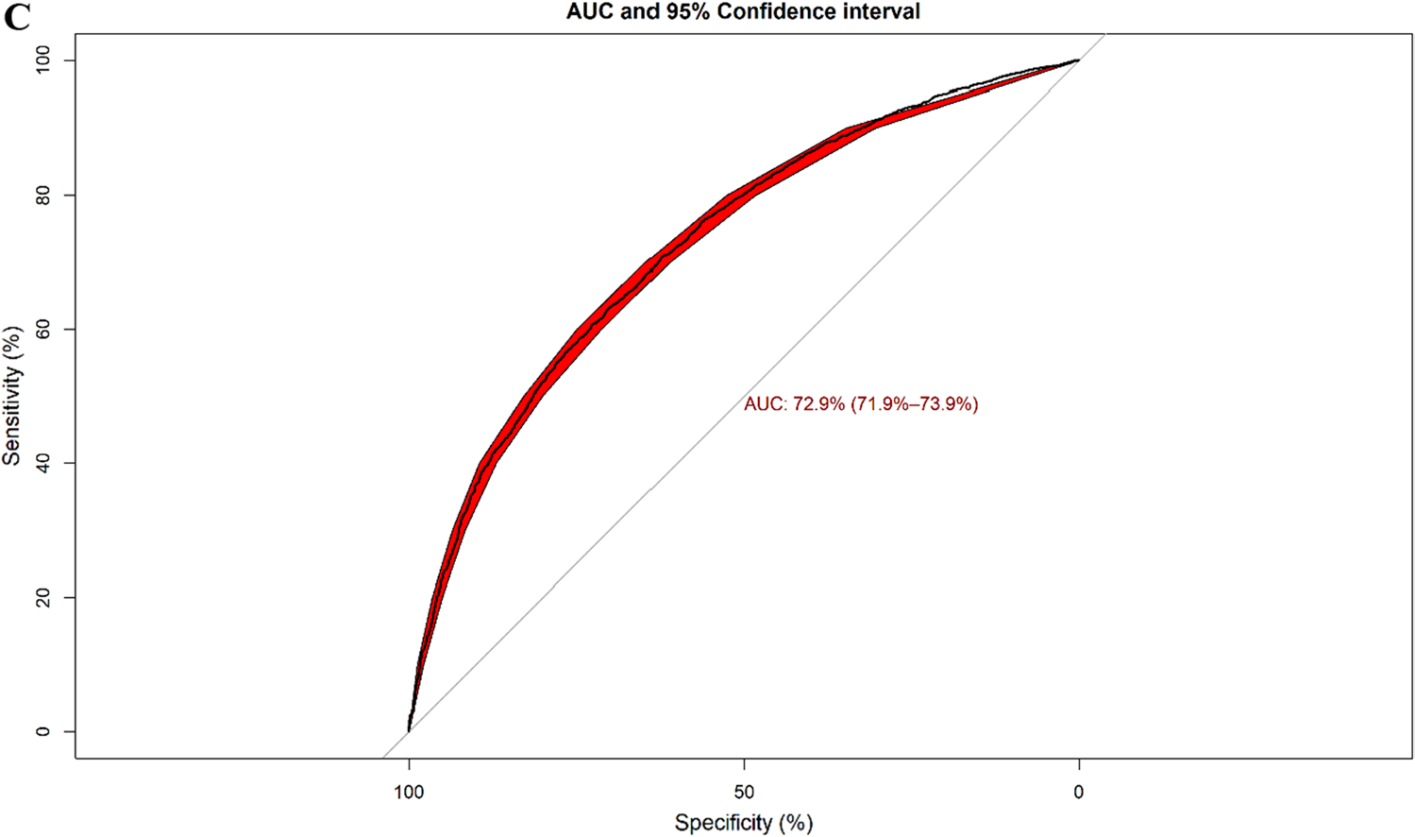
(**A**) ROC curve for all selected features by Elastic net-logistic regression classifier for predicting mortality risk based on the testing set (AUROC: 73.35%, 95% C.I 72.25–74.36%). (**B**) ROC curve for all selected features by SCAD-SVM classifier for predicting mortality risk based on the testing set (AUROC: 73.75%, 95% C.I: 72.64–74.75%). (**C**) ROC curve for MCP- neural network classifier for predicting mortality risk based on the testing set (AUROC: 72.95%, 95% C.I 71.85–73.95%).

Three subsets of the selected variables were used for binary classification of the mortality risk by LR, SVM, and NN with 100-times repetition in the testing set. Based on the classification metrics used for evaluating the predictive performance, the classifiers showed an approximately similar overall performance (Table 3). Accuracy (ACC), balanced error rate (BER), AUROC, sensitivity, and specificity were calculated based on the confusion (or classification) matrix for each classifier. The Elastic-net logistic regression had average ACC, BER, AUROC, sensitivity, and specificity of 70.09%, 29.91%, 73.35%, 63.58%, and 73.83%, respectively. The same metrics for the SCAD-SVM were 70.77%, 29.23%, 73.75%, 64.36%, and 74.0%, respectively. In addition, these metrics for MCP-NN were 69.59%, 31.41%, 72.95%, 63.03%, and 71.96%, respectively. According to these results, the predictive performance of the SCAD-SVM was non-significantly better than others. The receiver operating characteristic curve (ROC) of different classifiers are provided in Fig. 3. The results of the DeLong test for statistical comparison of AUROCs between the classifiers showed no significant difference among the three classifiers based on their AUROC values (*P*-value = 0.249, SVM vs. NN; *P*-value = 0.498, LR vs. NN; *P*-value = 0.816, LR vs. SVM).

**Table 3:** Comparisons of median classification indices between algorithms based on the selected features related to mortality status using elastic net-penalized logistic regression (“glmnet” R package), SCAD-penalized support vector machine with Sigmoid Kernel (“penalizedSVM” R package) and MCP- neural networks (“neuralnet” R package) repeated 100 times in the testing set (N = 9341).

Algorithm	Number of selected variables	ACC	BER	AUROC	Sensitivity	Specificity
Elastic-net logistic regression	38	0.7009	0.2991	0.7335	0.6358	0.7383
SCAD-SVM	25	0.7077	0.2923	0.7375	0.6436	0.740
MCP-neural network	40	0.6959	0.3141	0.7295	0.6303	0.7196

ACC: accuracy, AUROC: area under receiver operating characteristic curve, BER: balanced error rate.

### Survival modeling

The median survival time from diagnosis of dementia was 1096 (IQR = 566–1771) days and the censoring (i.e., surviving) rate was 33.71%. Among the twenty selected variables by all three algorithms, 15 variables were identified as highly important variables to predict time from diagnosis to death using the multivariable CoxBoost model. Among these variables, age at diagnosis (23%), MMSE score (15%), sex (14%), BMI (12%), and Charlson Comorbidity Index (CCI) (10%) were the most frequently selected by the CoxBoost model. All fifteen variables had significant effects on survival time among the patients, except atorvastatin and statin prescription in the year preceding dementia diagnosis where the *p*-values did not reach statistical significance. A higher age at diagnosis significantly increased the hazard rate of mortality (hazard ratio (HR) = 1.059, 95% CI 1.056–1.063). In contrast, higher BMI, and MMSE score significantly decreased the hazard rate of mortality in patients (HR = 0.967, 95% CI 0.962–0.974 and HR = 0.945, 95% CI 0.941–0.949, respectively). Taking diuretics (HR = 0.692, 95% CI 0.657–0.729) and cholinesterase inhibitors (HR = 0.724 95% CI 0.689–0.761) significantly decreased the hazard rate of mortality in these patients (Table 4). The C-index and Gonen and Heller's Concordance Index (GHCI) of the CoxBoost model were 0.6987 and 0.6682, respectively indicating an acceptable fit.

**Table 4:** Multivariable survival analysis for assessing the effects of the twenty-selected variables by the CoxBoost modeling (“CoxBoost”R package) with holdout validation (2/3 training and 1/3 testing samples).

Selected variable	HR (95% C.I)	*P*-value	Adj. *P*-value	Selection frequencies	C-index	GHCI
Age at dementia diagnosis	1.059 (1.056–1.063)	< 0.00001	< 0.00001	23%	0.6887	0.6682
MMSE score	0.945 (0.941–0.949)	< 0.00001	< 0.00001	15%		
BMI	0.967 (0.962–0.974)	< 0.00001	< 0.00001	12%		
Basic dementia diagnostic work-up	NA	NA	NA	NS		
Time from referral to initiation of work-up (days)	1.001 (1.001–1.002)	< 0.00009	< 0.001	3%		
Time from initiation of work-up to diagnosis (days)	1.001 (1.001–1.001)	< 0.00001	< 0.00001	2%		
Physiotherapist assessment (no-ref.)	NA	NA	NA	NS		
Diuretics (no-ref.)	0.692 (0.657–0.729)	< < 0.00001	< 0.00001	6%		
Charlson comorbidity index	1.141 (1.127–1.154)	< 0.00001	< 0.00001	10%		
Atorvastatin (no-ref.)	0.938 (0.829–1.060)	0.305	0.305	1%		
Rosuvastatin (no-ref.)	0.645 (0.473–0.881)	0.005	< 0.01	1%		
Statins (no-ref.)	0.959 (0.911–1.011)	0.121	0.211	2%		
Sex (female-ref.)	1.252 (1.191–1.314)	< 0.00001	< 0.00001	14%		
Heart failure (no-ref.)	NA	NA	NA	NS		
Cholinesterase inhibitors (no-ref.)	0.724 (0.689–0.761)	< 0.00001	< 0.00001	6%		
Total number of medications at the time of diagnosis	NA	NA	NA	NS		
Liver failure (no-ref.)	NA	NA	NA	NS		
Diabetes mellitus (no-ref.)	1.298 (1.215–1.387)	< 0.00001	< 0.00001	1%		
renin-angiotensin system inhibitors two or more years before dementia diagnosis (no-ref.)	1.246 (1.185–1.310)	< 0.00001	< 0.00001	3%		
Cancer (no-ref.)	1.311 (1.245–1.379)	< 0.00001	< 0.00001	1%		

HR: Hazard Ratio, 95% CI 95% Confidence Interval, adj. *P*-value: adjusted *P*-value by Benjamini–Hochberg Procedure, Ref.: reference level, selection frequencies were calculated by the cox-boost model, NS: not selected by the cox-boost model, NA: not applicable, GHCI: Gonen and Heller's Concordance Index, C-index: Chambless and Diao’s estimator of cumulative/dynamic AUC for right-censored time-to-event data, Number of boosting iterations in the CoxBoost model was 100.

### Clustering dementia patients based on the selected variables

**Figure 4:**
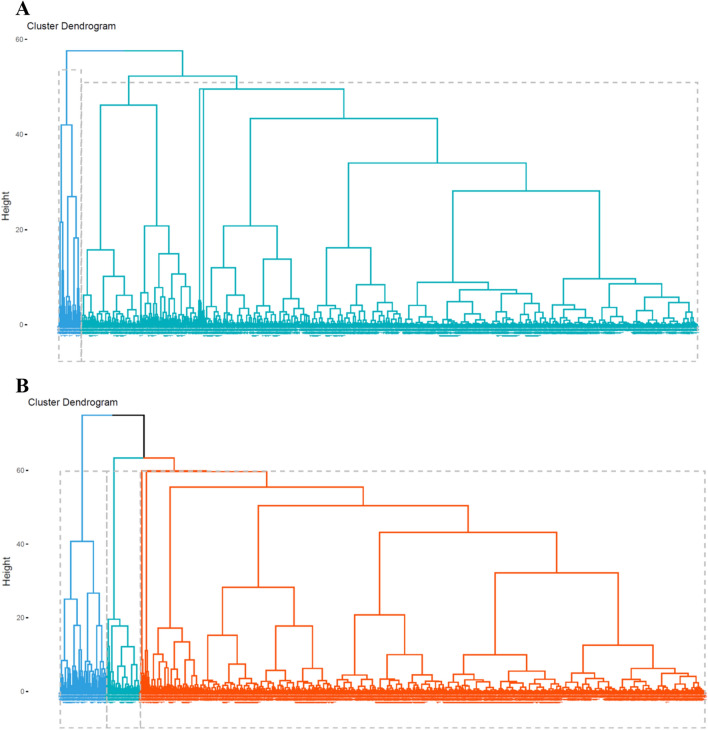
(**A**) Hierarchical clustering of the surviving patients (n = 9447), (**B**) Hierarchical clustering of the dead patients (n = 18,576) based on the twenty-selected variables to predict mortality risk.

**Figure 5:**
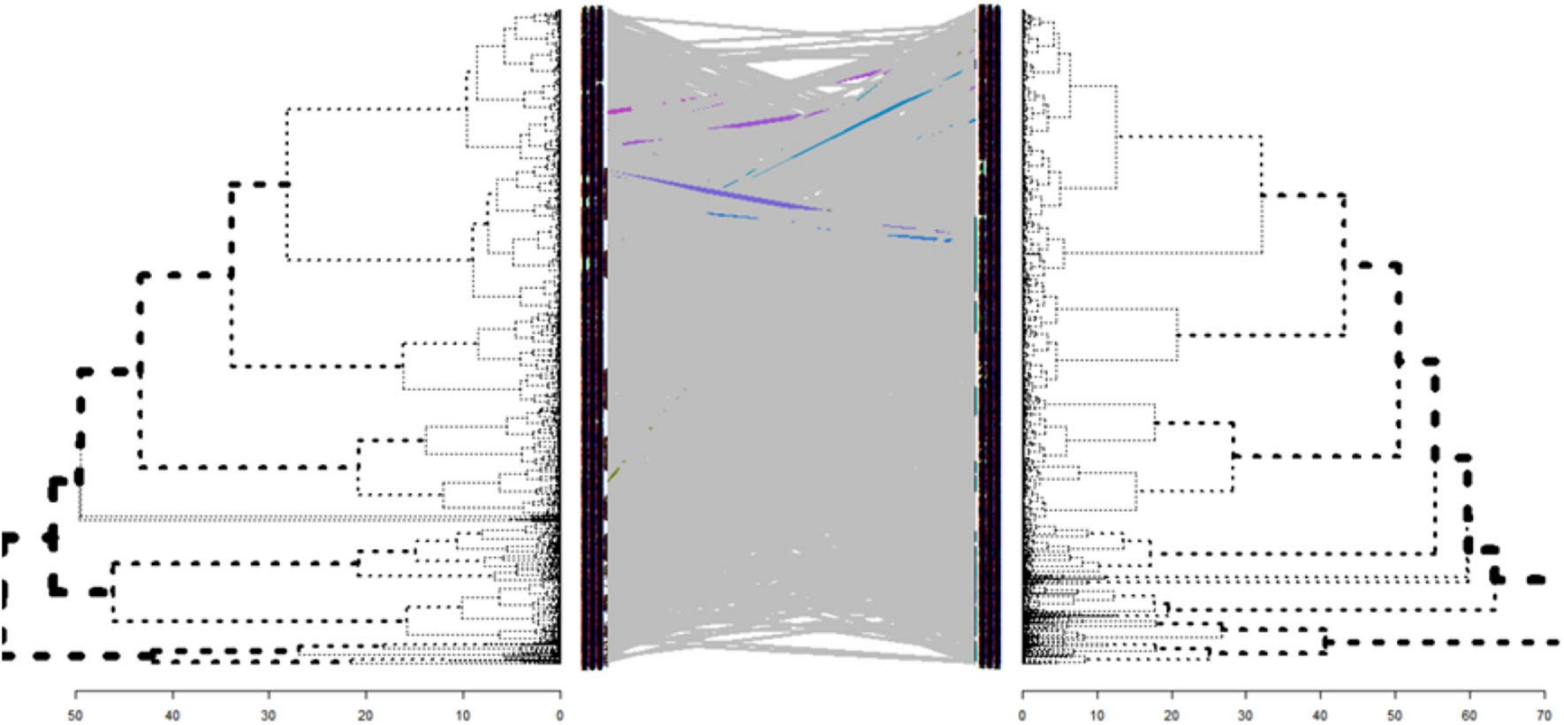
Statistical comparison of dendrogram between surviving-patients (right side) and dead-patients (left side) (Cophenetic correlation coefficient = −0.00018). This coefficient indicated that there is no similarity between dead and surviving-clusters based on the twenty-selected variables.

The Rand index was calculated to assess discrimination power of the classification models using the unsupervised hierarchical clustering algorithm. Based on similarities in the twenty selected variables associated with the mortality risk, Rand index was 0.63 and matched well with surviving and dead patient clusters. According to the results of the hierarchical clustering, dead and surviving patients, two major clusters among surviving patients and three major clusters in dead patients were identified (Fig. 4). Based on the height of dendrograms, heterogeneity among dead patients was higher than surviving patients. In more detail, there were significant differences in age at diagnosis, BMI, and MMSE score among the three clusters of dead patients (*P*-values < 0.001). There was no significant difference between both clusters of surviving patients based on the identified variables. The optimal cut-point of dendrograms to find the number of clusters was done by maximizing the variability of the observations between clusters. The comparison of the dendrograms between dead and surviving patients showed that there was no similarity or correlation between dead and surviving patients based on the twenty selected variables (Cophenetic correlation coefficient = −0.00018). Therefore, the results of the clustering confirmed that these selected variables could discriminate between dead and surviving patients overall. (Fig. 5).

### Sensitivity analysis

A post-hoc sensitivity analysis was performed to evaluate the robustness of our findings and assess the impact of missing data. This analysis involved examining the effect of different imputation methods and assumptions on the results, including last observation carried forward and locally weighted scatterplot smoothing. The results of the sensitivity analysis revealed variations in AUROC values across different imputation methods and somewhat improved AUROC values relative to the complete-case analysis. However, there is no guarantee to ensure that imputation analyses are unbiased. Eventually, the complete-case analysis was reported as the main finding in this study due to its simplicity.

## Discussion

In this large national cohort study, three standard classification algorithms were applied and evaluated. These algorithms used different sparsity-inducing penalties to identify the most important variables associated with mortality risk in PWD. The study aimed to identify previously unsuspected variables which were associated with mortality risk in these patients, to rank them in order of importance, and to develop models to predict mortality risk. We found that the diagnostic model generated by SCAD-SVM achieved a greater predictive performance but the differences were not significant between SCAD-SVM with MCP-NN and Elastic-net LR. The twenty selected variables were the same in all three algorithms. Both SVM and NN are margin-based classifiers and can model non-linear decision boundaries. LR had a similar classification performance in our study. A previous study found LR to have similar classification performance to NN and SVM in predicting diabetes risk^19^. The predictive power of SCAD-SVM in our study was AUROC = 0.7375 which is higher than the value calculated in another study in PWD^20^. Results of the survival analysis by the CoxBoost model showed fifteen variables further selected as highly important predictors for time to death in PWD. Our results from the survival model (i.e., C-index = 0.6987) are consistent with previous cohort studies on large-scale populations and our previous work on SveDem. Previous studies on SveDem showed that age, sex, residency, population density, comorbidity burden, MMSE score, BMI, number of medications used, and certain specific medications were significantly associated with time to death using Cox-Proportional Hazards model which C-index was between 0.65 and 0.72^7,8,10,11,17,18^. According to the comparison of the dendrograms between dead and surviving patients, there was no correlation between dead and surviving clusters based on the similarities of the selected variables (Cophenetic correlation coefficient = −0.00018). This means that these variables (i.e., age at diagnosis, BMI, and MMSE score) were significantly different between dead and surviving patients. This unsupervised clustering algorithm confirmed the discrimination power, validated the findings of the classification algorithms, and strengthened the results.

Classification and prediction models play significant roles in data analysis to build a diagnostic or prognostic model. There are many algorithms for classification and prediction tasks in the machine learning field. Among them, SVM and NN are two standard algorithms for classification in many situations (e.g., handling nonlinear classification and high-dimensional data)^21,22^. The NN algorithm is based on a more powerful and adaptive nonlinear equation form and can learn complex functional associations between the input and output data^23^. As a classifier, LR is much more popular than SVM and NN classifiers because it is easier to interpret. However, achieving a high prediction performance is not the primary goal, rather, identifying the most relevant variables is often the primary computational question. Therefore, variable selection methods (e.g., regularization) could be of great help by automatically connecting with many classification algorithms to avoid overfitting^24,25^. Variable selection methods can achieve the best subset of the most relevant variables for prediction and classification. As an important phase of classification and prediction, variable selection also improves predictive power while avoiding overfitting. Wrapper methods evaluate subsets of variables by training and testing the model on different combinations of variables. Wrapper methods are computationally expensive because they involve training and testing the model multiple times for different variable combinations. They are often used when the number of variables is relatively small. On the other hand, embedded methods are a group of variable selection methods carrying out variable selection within learning classifiers to achieve better computational efficiency and performance compared to wrapper methods. In other words, embedded methods are less computationally expensive and less prone to overfitting than the wrapper methods^26^. Regularization methods are effective embedded variable selection methods that provide an automatic variable selection within learning classifiers (e.g. LR and SVM)^27,28^. With different penalties, several sparsity variable selection methods can be applied. LASSO as the L_1_-norm penalty is considered as one of the most popular procedures in the class of sparse penalties. However, the result from this penalty is inconsistent. To overcome this limitation, Elastic-net penalty, as a convex combination of the LASSO and ridge penalty, can be helpful. Experiment and simulation studies have demonstrated that the Elastic-net often outperforms the LASSO for variable selection in classification task^28,29^. The MCP is very similar to the SCAD penalty. Both MCP and SCAD are non-convex or concave and enjoy the oracle property and unbiased estimates^30^. MCP performs well when there are many rather sparse groups of predictors. The main limitation of MCP and SCAD is when the non-zero coefficients are clustered into tight groups; as they tend to select too few groups and make insufficient use of the grouping information^31^.

Using different machine learning algorithms, we found that sociodemographics, cognition as measured by MMSE, comorbidities and drug utilization were the most important predictors of mortality risk. It is perhaps simpler to compare the results from the survival algorithm (i.e., CoxBoost) which most closely resemble the published literature using Cox-proportional hazards regression. We observed that high age, male sex, low BMI and MMSE predicted mortality risk of PWD. This result was in line with previous studies using data from SveDem, in which higher BMI was significantly associated with lower mortality risk^6^. This “obesity paradox” or reverse epidemiology has frequently been described in dementia and other conditions^32^. Preceding studies also showed that living situation was associated with mortality risk of PWD^18^. Unsurprisingly, higher MMSE score was significantly associated with lower mortality risk, as consistently shown in prior studies on SveDem and other cohorts^4,33,34^. In a previous study on SveDem, MMSE score was a significant predictor of mortality with HR = 0.964 (95% CI 0.962–0.967) per point of MMSE (≈4% risk decrease) among PWD registered in primary care and HR = 0.952 (95% CI 0.949–0.955) among PWD registered in a memory clinic^18^. This effect size is the same as the one calculated here with the Boosted Cox model (HR = 0.945; 95% CI 0.941–0.949). Comorbidities which were significantly associated with time to death in this study included diabetes and cancer. PWD with higher CCI also had significantly higher mortality risk with an effect size similar to the one reported by traditional multivariable Cox-proportional hazard regression performed on this same cohort^18^. However, previous studies found that mortality risk of PWD was higher among PWD suffering stroke^35–37^, which was identified as an important predictor for mortality by the Elastic-net LR algorithm. Despite some of these variables being familiar from previous research, the order of importance was sometimes surprising, for example, the high importance of BMI relative to other predictors.

Regarding drug utilization among PWD, we observed that the use of diuretics or rosuvastatin (but not statins in general or atorvastatin) was significantly associated with lower mortality risk. In another recent study using SveDem data, incident users of statins had a significantly lower risk of all-cause death (HR = 0.82, 95% CI 0.74–0.91) compared to non-users^38^. That study was propensity-score matched and included a somewhat older cohort which might explain the discrepancy. The use of diuretics might reflect comorbidities (e.g., hypertension) which could explain the association. We also found that consuming renin-angiotensin system inhibitors two or more years prior to dementia diagnosis significantly increased mortality risk of PWD, which is at odds with our expectations. It is possible that in this patient selection, the chronic use of renin-angiotensin inhibitors was confounded by the indication for treatment. Different types of drug utilization previously associated with mortality risk of PWD include glucose-lowering drugs, cholinesterase inhibitors, antipsychotics, anticholinergics, atrial fibrillation medications, and antidepressants^17,39–44^. The total number of medications at time of diagnosis and number of dementia medications were identified by all three algorithms matched our previous studies^4,17^.

Dementia type was identified by the Elastic net-LR and MCP-NN as a significant and important predictor of mortality risk and was also a strong predictor of mortality risk and survival time in our previous studies^4,18^.

What is most interesting is that the ML algorithm detected variables which we had not thought to explore in our cohort (factors without an “a priori” or pre-specified hypothesis). This was the case for the time between referral and initiation of diagnostic work-up and time from diagnostic work-up to diagnosis. These variables were identified by the algorithm with 3% and 2% of selection frequencies, respectively, and warrant further examination in future studies since they suggest a deleterious effect of long waiting lists on survival. The C-index for the CoxBoost model was 0.69 showing acceptable calibration in the testing set. However, a prior study from SveDem using forward selection of covariates arrived at a C-index of 0.705 including age, MMSE score, CCI score, dementia type, sex, living situation, and drugs in a Cox-proportional hazards model^18^. The clinical utility of this study lies in identifying several new predictors associated with mortality risk and which are potentially modifiable, since they are related to waiting lists. Also interesting is the ranking of predictors in order of importance, which can potentially help prioritize interventions and identify patients at risk. This may be a starting step to developing an individual model for each patient, as part of personalized medicine.

The most notable strength of this study was the large size of studied cohort and linkage of national registers. SveDem is the largest clinical dementia register in the world^45,46^. In addition, the Swedish National Patient Register (NPR) was also employed which covered all inpatient and specialist medical diagnoses. Furthermore, the data on dementia subtypes from SveDem was a unique feature of this study. Using different linear and non-linear ML algorithms, reducing omitted-variable bias by application of three different sparsity-inducing penalties and confirmation by an unsupervised clustering algorithm are other advantages of this study. However, there are some limitations that should not be neglected. Missing data is a weakness of this register-based study. Due to the high number of included predictors, only 28,023 patients (out of 80,004 PWD registered in SveDem) had complete data on all sixty potentially associated variables. We conducted a sensitivity analysis with different methods of imputation. We chose to keep the complete-case analysis as the main finding of the study because of the high percentage of imputed values and because the assumption for the imputation were not met, which could introduce bias. The NPR includes all inpatient medical diagnoses and outpatient care in Sweden but does not cover diagnoses in primary care. Thus, the prevalence of diseases, as well as the influence of Charlson Comorbidity Index on the algorithms might have been underestimated here. Moreover, the Swedish Prescribed Drug Registry (PDR) covers all prescription drugs sold in pharmacies in Sweden but does not include over-the-counter drugs or those administered during hospitalization.

## Conclusion

In this national dementia cohort study (i.e., SveDem), we applied different standard ML classifiers with three sparsity-inducing penalties to consistently identify important variables associated with mortality risk. The ML algorithms not only replicated some of the previously known findings but also ranked variables by importance, showing that higher age, male sex, low MMSE and low BMI were the most important predictors of death. They also identified new important variables such as performance of basic dementia diagnostic work-up, time of referral to initiation of work-up, time of initiation of work-up to diagnosis, and the use of diuretics. This study highlights the value of employing ML algorithms as a valuable addition to our analytical arsenal. ML can complement traditional statistical methods, particularly when dealing with large-scale, sparse, and heterogeneous data. Overall, this study demonstrates the potential of ML algorithms in improving our understanding of mortality risk factors in patients with dementia and their potential application in clinical settings.

## Methods

### Study participants

The Swedish Registry for Cognitive/Dementia Disorders Registry (SveDem) is a national quality-registry established in 2007 with the aim to register all patients with dementia in Sweden at the time of diagnosis and conduct follow-ups to improve dementia diagnostics and care^47,48^. SveDem can be merged with other registries using the Swedish unique personal identification number. This study included 60 variables potentially related to mortality status from SveDem and other registries and selected from the literature and our clinical knowledge and understanding of the registries: information on the patient’s demographics, living arrangements, date of diagnosis, co-morbidities, and medications taken at the time of the dementia diagnosis (baseline). Medication usage history was obtained from the Swedish Prescribed Drug Registry (PDR). The PDR was established in July 2005 and contains data on all prescribed drugs dispensed at pharmacies in Sweden^49^. Comorbidities were obtained from the Swedish National Patient Registry (NPR) which covers data on health care episodes in inpatient and outpatient specialist care and includes four different groups of data; demographic/patient data, geographical data, administrative, and medical data^50^. The date of death was ascertained from the Swedish Cause of Death Registry until December 31, 2018. From 80,004 patients registered in SveDem between 2007 and 2018, we included 28,023 persons diagnosed with no missing data on any of the sixty potentially predictors for a complete case analysis (CCA) in the ML algorithms. To avoid selection bias, missing at random was checked (i.e., the chance of data being missing was unrelated to any of the predictors involved in our analysis). The TRIPOD statement was reported for good reporting of the developing and validating multivariable prediction models in this study (Supplementary Information).

### Exposures and outcomes

Based on the literature review, recommendations from the clinicians (SGP and ME), and omitting poor quality/bad implementation variables, sixty variables were considered as potential predictors of mortality in the ML algorithms. These variables included age at dementia diagnosis, sex, dementia types, BMI, MMSE score, living situation (alone vs with another adult), residency (at home vs nursing home), performance of the basic dementia diagnostic work-up, types of diagnostic units (primary vs specialist care), time from referral to initiation of work-up, time from initiation of work-up to diagnosis, dementia medications (e.g., cholinesterase-inhibitors, memantine), total number of medications taken at time of dementia diagnosis, Charlson Comorbidity Index (CCI), comorbidities, and some specific medications for chronic comorbidities (e.g., antihypertensive, statins). The basic dementia work-up is defined by the Swedish Board of Health and Welfare^51^ and includes a structured clinical interview, an evaluation of the physical and psychological situation of the patient, an interview with a knowledgeable informer, MMSE, clock test, blood analyses, and neuroimaging. The last four of these are included as variables in SveDem and combined into the variable “basic dementia work-up” to be followed as a quality indicator for care. The study outcome was all-cause death. Patients were followed from the dementia diagnosis date to death or the end of follow up (31 December 2018).

### Variable selection, classification and evaluation

For the variable selection process, different sparsity-inducing penalties were used to remove irrelevant or redundant variables. There are generally three main categories of variable selection methods: wrapper methods, filter methods, and embedded methods. Wrapper methods evaluate subsets of variables by training and testing the model on different combinations of variables. The wrapper methods are often used when the number of variables is relatively small due to being computationally expensive. Filter methods assess variables independently of the model and consider their correlations with the outcome variable. The main disadvantage of filter methods is that they ignore variable dependencies. Embedded methods incorporate the variable selection process into the model building algorithm itself. These methods typically use regularization techniques to select the importance of certain variables (e.g., Elastic-net)^52^. To avoid omitted-variable bias (OVB) (i.e., missing out any important variables), regularization methods as effective embedded variable selection methods were applied by three different penalties: Elastic-Net, Smoothly Clipped Absolute Deviation (SCAD), and minimax concave penalty (MCP). All these overcome the limitations of traditional variable selection methods; for example, stepwise logistic regression requires large sample sizes and is more computationally expensive than these methods. Elastic-net linearly combines L_1_ and L_2_ penalties, uniting the strengths of both Least Absolute Shrinkage and Selection Operator (LASSO) (L_1_) and ridge (L_2_)^28^. This is important because LASSO penalty is suitable for variable selection but not for group selection and it tends to give biased estimations. We suspected that our exposure variables were correlated and LASSO tends to select only one among correlated variables. So, group selection methods (e.g., Elastic-net) were important in our study^28^. Elastic-net penalty is suitable for multi-collinearity and grouped selection situations (like ridge-L_2_ penalty) and it has good performance for simultaneous estimation and variable selection (similar to LASSO-L_1_ penalty)^28^. Elastic-net penalty has a strictly convex loss function and, therefore, a unique solution/global optimum and parameter estimation (oracle properties)^28,53^. On the other hand, SCAD and MCP penalties are non-convex optimizations which means that there are more than one local optimum and they are computationally harder than LASSO or Elastic-net. Additionally, we can only obtain a local optimum with these penalties and not the global optimum. MCP, SCAD and Elastic-net all assign zero-coefficients to non-identified variables. SCAD and MCP have less biased estimates than Elastic-net for the non-zero coefficients, i.e. the selected variables^54^. Moreover, MCP’s advantage over SCAD is giving less biased coefficients in sparse models^55^. Both MCP and SCAD penalties outperform Elastic-net based on their less biased estimation of the coefficients while Elastic-net has the advantage of giving a unique parameter estimation. MCP and SCAD penalties suffer when the identified variables are clustered into tight groups as they tend to select too few groups and make insufficient use of the grouping information^31^. All these penalties have some tuning parameters. Estimation of the best value for tuning parameters is important to decide how many variables are to be selected. We applied 100-times repeated 10-fold cross-validation technique to estimate the tuning parameters and establish consistency in the variable selection processing in the training set^53^.

For the binary classification of mortality risk, we used three standard classifiers including LR, SVM, and NN. LR is one of the most common classifiers used in epidemiological studies and is based on a linear decision boundary. When non-linear relationships exist, a nonlinear decision boundary may result in better overall performance. SVM and NN are designed to generate more complex decision boundaries. In other words, both classifiers can detect nonlinear relationships between outcome and predictors. SVM (e.g., sigmoid kernel) has the advantage of taking non-linear associations and mapping them into linear boundaries improving interpretability, whereas NN has several hidden layers and, hence, interpretation of its classification decision is difficult. NN requires more complex computations to train the algorithm compared with LR and SVM. SVM can include varying degrees of non-linearity and flexibility by using different kernel functions. Unlike LR and NN, classification results of SVM are purely dichotomous whereas LR and NN give a probability of class membership. Overfitting is less of an issue in LR because LR is less sensitive to training samples compared to NN and SVM algorithms. In contrast, NN is more complex and, thus, more susceptible to overfitting than LR and SVM^56^. To overcome this issue, regularization methods (i.e., sparsity-inducing penalties) could be helpful^56,57^. Finally, we used sigmoid kernel for the SVM and Softmax activation function with one hidden layer and 10 hidden neurons for the NN algorithm in this study. Each classifier was combined with all three penalties/regularization methods to perform variable selection and binary classification simultaneously. The importance values in each model were calculated based on the Gini index with normalization.

The final step in the mortality classification was to check for overfitting. This was done using the holdout method where all samples in the dataset were randomly divided into 66.6% (18,682 samples) and 33.4% (9341 samples) as training and testing sets, respectively. Accuracy (ACC), balanced error rate (BER), area-under-curve measure associated with receiver-operating-curve (AUROC), sensitivity and specificity were reported for the test set as the classification metrics of the performance on the testing samples. Statistical comparison of AUROCs among the different classifiers was performed by the DeLong test to identify the best algorithmic combinations of binary classifiers and sparsity-inducing penalties for the mortality risk prediction^58^. All statistical analyses were performed by “glmnet”, “penalizedSVM”, “neuralnet”, “ncvreg”, and “pROC” R packages^21,59–61^.

### Survival modeling

CoxBoost was used to develop a robust survival model based on the selected variables in all combinations of classifiers and sparsity-inducing penalties (i.e., Elastic net-LR, SCAD-SVM, and MCP-NN). This survival model can be applied to fit the sparse survival models and this enables us to consider some mandatory covariates in the model based on the likelihood-based boosting^62,63^. Previous studies have shown that CoxBoost has a high goodness of fit compared to a Cox proportional hazard model where there are many predictors; since it allows mandatory covariates with unpenalized parameter estimates^62,64^. Boosting is a popular iterative technique used in survival analysis with a high flexibility for the selection of the candidate variables and ease of interpretation. Boosting is also applicable in many situations where the assumption of proportional hazard (PH) does not exactly hold^65^. In our case, we used “CoxBoost” R package^66^. The model was trained by 2/3 samples (18,682 training samples) and tested on 1/3 samples (9,341 testing samples). The concordance index (C-index), as an evaluation metric of survival models, is a weighted average of the area under time-specific ROC curves (time-dependent AUC)^67^. The C-index and Gonen and Heller's Concordance Index (GHCI) were reported to assess the performance of the survival model in the testing set^68^.

### Hierarchical clustering

To validate the identified variables by an unsupervised clustering algorithm, agglomerative hierarchical clustering and Rand index were applied to assess discrimination power of the classifiers that match well with surviving and dead patient clusters^69^. For clustering of the patients in surviving and dead groups, the data were divided into two datasets of surviving and dead patients. Then, the agglomerative clustering algorithm was run separately on each dataset to identify clusters of the patients based on the similarities in the identified variables. The clustering results for the surviving and dead patient groups were compared to confirm the presence of considerable differences based on the identified variables between dead and surviving patients. More technically, this hierarchical clustering algorithm was performed by “binary” distance measure and the “ward.D2” method. We compared dendrograms in dead and surviving clusters by the Cophenetic correlation coefficient and permutation test/10-times^70^. The “cluster”, “dendextend”, and “factoextra” R packages were applied for clustering, comparison of dendrograms, and visualization, respectively^71^.

**Figure 6:**
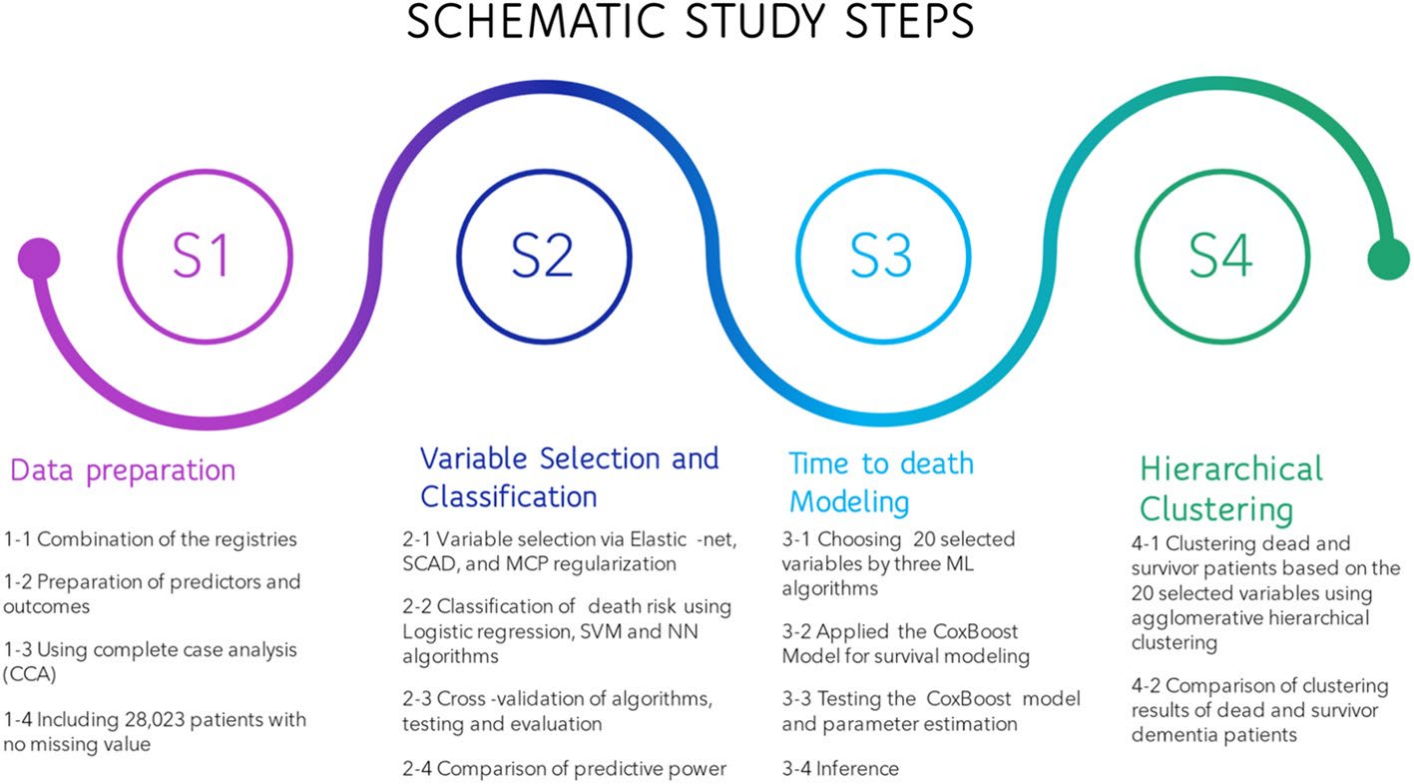
The flowchart of this study represents the different machine learning steps.

All statistical analyses were performed using R software version 4.1.1 (The R Foundation for Statistical Computing). The significant level was considered at a level of 0.05. Figure 6 summarizes the different computational steps adopted in this study.

### Ethical approval and consent to participate

This project was approved by the Swedish Ethical Review Authority with the reference number (#2021–0043) and was performed based on the Declaration of Helsinki guidelines. Patients were informed about registration in SveDem at the time of their dementia diagnosis and gave informed consent to obtain information on their registration any time and could withdraw consent later. Data were de-identified by Swedish authorities before delivery to the research team.

## Supplementary Information

Supplementary Information.

## Data Availability

The data are not available for public access following Swedish and EU legislation. Researchers may apply to obtain data from Swedish registries after obtaining ethical approval, following the standard rules and regulations, and applying to the steering committees of the registries and to the relevant government authorities.

## Abbreviations

ML: Machine learning
SveDem: The Swedish Registry for Cognitive/Dementia Disorders
BMI: Body mass index
MMSE: Mini-mental state examination
AUROC: Area-under-receiver operating characteristic curve
SVM: Support-vector-machines
LR: Logistic regression
NN: Backpropagation neural networks
SCAD: Smoothly clipped absolute deviation
MCP: Minimax concave penalty
CCI: Charlson comorbidity index
PWD: Patients with dementia
COX PH: COX proportional hazard
PDR: Swedish prescribed drug registry
NPR: Swedish national patient registry
CCA: Complete case analysis
LASSO: Least absolute shrinkage and selection operator
ACC: Accuracy
BER: Balanced error rate
GHCI: Gonen and Heller's Concordance Index
IQR: Interquartile range
HR: Hazard ratio
95% CI: 95% Confidence interval
